# Informal Caregivers Connecting on the Web: Content Analysis of Posts on Discussion Forums

**DOI:** 10.2196/64757

**Published:** 2025-01-17

**Authors:** Michelle L Foster, Chinenye Egwuonwu, Erin Vernon, Mohammad Alarifi, M Courtney Hughes

**Affiliations:** 1 School of Health Studies, Northern Illinois University, DeKalb, IL, United States; 2 Department of Internal Medicine, Piedmont Athens Regional, Athens, GA, United States; 3 Department of Economics, Seattle University, Seattle, WA, United States; 4 Radiological Sciences Department, College of Applied Medical Sciences, King Saud University, Riyadh, Saudi Arabia

**Keywords:** informal caregivers, family caregivers, discussion forum, caregiver support, support group, social support, caregiver navigation, content analysis, adults, United States, informal care, codebook, thematic analysis, web-based discussion, web-based forums, clinicians, medical care, peer-to-peer support, web-based communities, caregivers

## Abstract

**Background:**

About 53 million adults in the United States offer informal care to family and friends with disease or disability. Such care has an estimated economic value of US $600 million. Most informal caregivers are not paid nor trained in caregiving, with many experiencing higher-than-average levels of stress and depression and lower levels of physical health. Some informal caregivers participate in web-based forums related to their caregiving role.

**Objective:**

This study aimed to explore how informal caregivers use easy-to-access caregiving web-based forums, including the types of information they share and seek from others. It also aimed to gain insights into the informal caregiver experience from the content these informal caregivers posted.

**Methods:**

The study population consisted of participants who posted on 5 web-based forums for informal caregivers between February and April 2024. Researchers extracted the first 6 responses to the first 20 questions and comments to appear posted by the informal caregivers in each of the 5 forums, removing any individually identifying information. We used a codebook thematic analysis approach to examine the data with Dedoose (SocioCultural Research Consultants). Researchers independently read all posts and coded the data. The author group discussed the codes, reiteratively refined them, and identified themes within the data.

**Results:**

The data consisted of 100 initial posts and 600 responses. Over half of the initial posts included specific questions, with the remaining initial posts sharing experiences or reflections. Posts ranged in length from a sentence to more than 500 words. Domains identified included handling interpersonal challenges, navigating complicated systems, gathering tactical coping strategies, managing emotions, and connecting with others in similar situations. Negative interpersonal interactions were mentioned 123 times, with 77 posts describing challenging situations with extended family. Posters inquired about accessing resources, with health care and health insurance included 51 times, while legal and financial concerns were addressed 124 times. Caregiving challenges were mentioned hundreds of times, including discussion of hygiene (n=18), nutrition (n=21), and desire for a caregiving break (n=47). Posters expressed emotion in their comments 180 times, which included 32 mentions of guilt and 26 mentions of positive emotion. The importance of web-based group support was mentioned 301 times.

**Conclusions:**

Informal caregivers play an essential role in society. Many experience multifaceted challenges related to their caregiving role, and some turn to the internet for community. Accessing web-based discussion forums is a low-barrier method for informal caregivers to connect with others who may be experiencing similar emotions and challenges. Gaining a greater understanding of the ways informal caregivers seek advice and offer support to one another provides insight into the challenges they face. The domains identified on these forums may be helpful, as clinicians provide information to care recipients and their informal caregivers along their health journeys.

## Introduction

About 53 million informal caregivers assist individuals in need of medical care and activities of daily living in the United States [1]. The unpaid value of this care was about US $600 billion, up from a 2017 estimate of about US $470 billion value of unpaid informal care. Informal caregivers are generally unpaid and untrained, and historically, these caregivers performed household tasks and personal care activities in contrast to formal caregivers who were part of the health system [2]. Research shows that more than 58% of informal caregivers are responsible for medical or nursing tasks for their care recipients [3].

Informal caregiving is associated with both benefits and burdens for caregivers. Many informal caregivers experience higher levels of stress and depression and have lower levels of physical health than their counterparts [4-6]. Care recipients with poor levels of physical and mental health were associated with worse caregiving experiences [5,6]. Furthermore, informal caregivers with older age or mental health issues have been shown to have worse caregiving experiences [5,7].

Most informal caregivers have internet access and use it to obtain health information [8]. There is significant research regarding the use of web-based tools among informal caregivers [9-18]. Some research used web-based surveys to ask about informal caregiver needs [10,11,17], while others evaluated the use or content of various purpose-built social support service interventions [13-15,18]. The qualitative research in this space has focused on specific topics related to caregiving, such as well-being during COVID-19 [19], advance care planning [20], and developing the identity of an informal caregiver [21]. Many other qualitative analyses have focused on particular diagnoses, such as care recipients with Alzheimer disease or dementia [22-25], posttraumatic stress disorder [26], stroke [27,28], and cancer [29-31]. Qualitative research about general topics addressed in informal caregiver forums focused only on 1 discussion platform (Reddit) and was evaluated with the use of artificial intelligence [32].

The previous studies investigating informal caregivers’ use of web-based forums have found that informal caregivers use web-based support groups and forums to find emotional support [13-15,19,20,22-28,30], request information [14,20,23,26-28,30], discuss symptoms and conditions [23,24,27,28], interact with other caregivers [13,15,19-21,23,26,30], and talk about the challenges of caregiving [15,19,20,22,23,25,26] ]. Additionally, all existing studies but one [27] evaluated data from 1 specific web-based forum rather than more than 1 forum. The study herein aims to help fill the gap in research that examines how informal caregivers use caregiving web-based forums that are not restricted to just 1 forum. This is also the first study to examine the forums that appear first when a caregiver searches for chronic illness caregiver support forums on Google. This approach can shed light on how caregivers interact with one another across multiple caregiving web-based forums.

This study explores how informal caregivers used a variety of popular, easy-to-access web-based forums. Specifically, we sought to discover the types of information individuals posting to web-based informal caregiver forums shared and sought from others. Insights into the informal caregiver experience from exploring multiple discussion forums can inform health care providers and other community groups so they may better design interventions and policies responsive to caregiver needs.

## Methods

### Study Population

The study population consists of participants who posted on publicly accessible web-based forums for informal caregivers between February and April 2024. Descriptions of the forums (AgingCare [33], Caregiver Action Network [34], Facebook Dementia Caregivers Support Group [35], Facebook Empowering Caregivers Support Group [36], and Mayo Clinic Caregivers Forum [37]) are presented in Table 1. We selected these forums because they appeared as the top 5 forums in a web-based Google search using the phrase “caregiver support forum and chronic illness.”

**Table 1.** Description of web-based caregiver forums from each forum’s website.

Forum name	Description	Details
AgingCare [33]	“Connects families who are caring for aging parents, spouses, or other elderly loved ones with the information and support they need to make informed caregiving decisions.”	Caregivers seek mutual support for medical challenges that occur with aging such as dementia Caregivers offer valuable suggestions based on firsthand experience managing these conditions
Caregiver Action Network^a^ [34]	“The nation’s leading family caregiver organization working to improve the quality of life for the more than 90 million Americans who care for loved ones with chronic conditions, disabilities, or disease, as well as those supporting the living needs of older adults.”	Caregivers seek support for general, day-to-day issues in caregiving Caregivers share personal experiences while caring for their loved ones with disabilities or disease
Facebook Dementia Caregivers Support Group [35]	“A group for caregivers taking care of Dementia and Alzheimer’s afflicted loved ones. Designed to stimulate conversation and ensure all members a safe haven in posting personal feelings.”	Caregivers seek support for general challenges with caregiving Caregivers seek support for caring for loved ones with neurocognitive behavioral changes and degenerative diseases such as delirium and dementia
Facebook Empowering Caregivers Support Group [36]	“A public group created so that there is a place for family members to turn to when in need of mental, emotional, and informational support when it comes to taking care of aging family members.”	Caregivers seek support for the day-to-day challenges of aging family members Caregivers seek support on specific information such as health system challenges and legal issues
Mayo Clinic Connect [37]	“An online community, connecting patients and family caregivers with each other. You do not have to be a Mayo Clinic patient or caregiver to join the conversations.”	Many forum members are caregivers of patients with chronic medical conditions, such as cancer, who receive treatment at the Mayo Clinic, though this is not required Caregivers typically seek support for medical challenges, such as medication side effects

a Caregiver Action Network’s caregiver forum was on its website at the time of the research. It has since moved to Facebook.

### Data Analysis

Two researchers (MA and CE) extracted the first 6 responses from the first 20 questions and comments to appear posted by the caregivers in each of the 5 forums. We chose this method based on the passive or unobtrusive method, which involves techniques that allow researchers to collect data without actively engaging with participants or influencing the conversation [38]. We did not extract questions and responses that did not contain comments relating to caregiver support (spam posts) because they were irrelevant to the research topic. To protect the identity of all posters on the web-based forum, we removed any names that appeared in the comments.

We used a subjective approach to gain insights into the caregiver forum participants’ perceptions of their experiences. Specifically, we adopted an approach based on grounded theory using an iterative inductive process for our qualitative thematic analysis. As such, the number of comments and posts collected were adjusted iteratively until 2 authors (MLF and CE) separately believed that thematic saturation within forums’ comments and posts was reached [39]. Similarly, the authors began with 2 forums and then added the next highest publicly accessible forum listed on Google until they achieved thematic saturation across forums. Using a codebook thematic analysis approach to examine the data [40] with Dedoose (SocioCultural Research Consultants), 2 researchers (MLF and CE) read through all the posts and coded the data. The entire author group discussed the codes, refined them reiteratively, and identified themes within the data. The author group also resolved any coding disagreements and resulting frequencies of each theme through discussions and collective agreements. They also examined the data to ensure that the chosen themes accurately reflected the data. Additionally, our author group comprised researchers with varying caregiving experiences. However, none of the researchers have intense caregiving experience that would deeply influence the research.

### Ethical Considerations

The institutional review boards at Northern Illinois University and Seattle University determined the study to be exempt from institutional review board review in accordance with federal regulation criteria. The data used in this study were collected from forums that are easy to access and generally open to the public. All data were deidentified before analysis, including removing usernames and other naming data to maintain anonymity and confidentiality. There was no contact between the researchers and the posters.

## Results

### Overview

The data consisted of 100 initial posts and 600 responses to the initial posts or conversations stemming from the initial posts. Over half of the initial posts included specific questions, while the others shared reflections or experiences. The length of the initial posts and responses varied from a few with just a short sentence to others that were a couple of paragraphs. A few stretched beyond 500 words—a full page of single-spaced text. Response lengths were similar, with ranges from a few words to more than 500.

Posters (our term for individuals posting anything, including initial posts and responses) to the forums discussed care recipients with various diseases and cited dementia and cancer the most. We found some variation in the care recipient’s disease based on the forum used. The Mayo Clinic Caregivers Forum included more clinical posts, with posters sharing specific disease states. In the Facebook Dementia Caregivers Support Group, some posters asked about what medications had worked for other care recipients and for strategies to obtain particular treatments, with almost all having to do with dementia. In the other forums, the care recipient’s diseases often were not apparent, with many posters omitting such details.

Initial posts often included significant details describing the poster’s life with their care recipient. Some posters responded with advice or feedback on the situation, while others shared their own experiences with their care recipients. Below, we present 5 key domains that resulted from the themes.

### Domains

#### Handling Interpersonal Challenges

In these forums, posters shared their varying daily interactions, ranging from toileting and feeding to medication management and household chores. These interactions sometimes led to interpersonal challenges with the person receiving care or others involved in the household, with such negative interactions recorded 123 times in these posts. One poster noted his care recipient wife was upset with how he did things around the house.

She is very exacting on how she wants things done. I am hard of hearing and she gets upset when she has to repeat herself. If I guess at what she wants and get it wrong, oh well, you get the picture. I have to control myself better and be there for her. I have to swallow my pride and do what is right for her. It is not easy*.*

[Mayo Clinic Connect]

In total, 10 posts described challenges related to changing relationship roles. Husbands and wives recounted working to feed their spouses and care for them throughout the day. The posters shared their feelings, which often included frustration and sadness about these changes.

I lost an equal parent, as well as my romantic and “relational” partner, partly. It’s a loss that is fully understood only by other spousal caregivers. It hasn’t gotten any easier after 6 years*.*

[Caregiver Action Network]

Some posters also described negative situations with extended family members, with such interactions reported 77 times. Posters shared that some siblings were only concerned about financial arrangements after death, while other siblings were described as expressing negative attitudes and overall dissatisfaction with care.

She is very difficult to deal with. She questions everything and does not trust me to care for our parents in a manner that is in THEIR best interest. Without details suffice to say my husband and I gave up our dreams to take care of my parents but she berates me, tries to put my other siblings against me, says I lie, and accuses me of taking things*.*

[Facebook Empowering Caregivers Support Group]

Other posters described a lack of family support for the care recipient and feeling isolated as the main caregiver. This was highlighted 33 times in these posts, with some posters, noting that they did not get breaks or have family visitors who could relieve the ever-present demands of caregiving. Other posters wrote that this constant responsibility negatively impacted their relationships with their own spouses and children.

My husband and kids are not getting the best version of me. I can’t stand having sex now because I spend so long caring for my parents the idea of sharing intimacy with my husband is actually repulsive to me. I don’t enjoy anything that I’m doing*.*

[AgingCare]

#### Navigating Complicated Systems

Posters asked and responded to questions about accessing resources related to health care, living arrangements, and financial and legal systems. They mentioned health care and health insurance systems in 51 posts. Other posters responded by sharing their experiences with the health care system as a guide for others and sometimes suggesting various social services. Information on accessing home health aides, hospice care, and various medicines was requested 47 times in posts. Ten discussed necessary steps for care recipients to qualify for nursing home access through Medicaid, such as spending down funds or putting assets into a trust.

I can’t wait until my mom is approved for Medicaid. She’s in the nursing home. We [are] just waiting on that. I spent all her money. Most of it went to the nursing home. Also when I sent information to Medicaid I sent copies of all checks to show where her money went.

[Facebook Dementia Caregivers Support Group]

I have been her sole caregiver for about 5 years and I know caring for her now is beyond my capabilities. What do people do when they can no longer keep their parent in the home? Money is a huge issue, her insurance doesn’t want to cover anything. I don’t know what to do at this point. She needs more than I can give her.

[Facebook Empowering Caregivers Support Group]

Posters expressed concern about the quality of care hospice or nursing home workers provided to their care recipients 23 times and asked for feedback on how to advocate. They described their situation and asked for advice on what to do when the nursing care was not working out as expected.

The nurses have asked that when I come to visit I help them get her to the shower ... I can’t be there all the time to make sure she gets up, takes a shower, just participates with life? The CNAs try to help, the nurses have told me that they can’t force her because they could get into trouble.

[AgingCare]

Posters responded with messages stating how they understand these caregivers’ concerns. They offered ideas on how they had dealt with similar situations and shared details about similar struggles they faced in working with care homes or hospice providers.

Posters described legal and financial concerns related to caregiving 124 times and asked others to weigh in on their options. Posters sometimes asked how to work within the legal system to help their loved ones. Some individuals receiving care wanted the informal caregiver to inherit a greater portion of assets upon their death, and posters asked about how to make the care recipient’s wishes clear. Respondents offered suggestions to these legal and financial concerns, often urging posters to consult with professionals.

If your mom is of sound mind and wants to make this change, that’s her decision. With the no-contest order and the doctor’s assessment, legal challenges from your siblings might not hold up. Considering your exhaustion, it might be worth seeking legal advice to prepare, but ultimately, your well-being is the priority.

[Facebook Empowering Caregivers Support Group]

#### Gathering Tactical Coping Strategies

Posters described hundreds of challenges related to their caregiving roles. They asked questions about how to address these concerns, and respondents offered ideas or shared how they had responded in similar circumstances.

One common theme was difficulties related to hygiene among those receiving care, highlighted in 18 posts. Posters described challenges related to body odors and getting care recipients to shower or bathe. One poster asked for help with their care recipient who was touching foods in their kitchen pantry, which caused the poster concern about contamination of foods due to dirty hands. Fellow posters pitched in with various ideas.

Find a lock for the pantry but leave snacks out for him on the counter that are just for him. My Mom goes prowling for snacks at night so we leave some out for her.

[Facebook Dementia Caregivers Support Group]

I keep extra bags of chips because I can’t eat out of the ones already opened. I have to constantly remind my [loved one] to wash her hands so I know if I’m not here they are probably not getting washed and she takes her dentures in and out of her mouth which totally grosses me out.

[Facebook Dementia Caregivers Support Group]

Concern about a loved one eating too much sugar or other nutrition concerns were mentioned in 21 posts. Respondents said that they experienced the same problems and highlighted that desire for sweets is common among people with dementia. Other posters responded with a variety of recipes and food ideas.

Posters expressed a desire for a break from caregiving duties 47 times. Respondents offered helpful tips, such as enlisting community or church volunteers. Enrollment in palliative or hospice care was mentioned in 55 posts, and posters shared their experiences engaging in those services.

It wasn’t until she was in home hospice that they were ever able conquer her incredibly high anxiety. We certainly realized no course of care is ever perfect and always knew palliative care was her only choice given her situation. We never regretted the care she received.

[Mayo Clinic Connect]

#### Managing Emotions

Posters expressed emotions in their comments 180 times. Some described themselves as being “stressed,” “overwhelmed,” “weary,” or “experiencing burnout.” They talked about their emotions, which often included anger, depression, sadness, and resentfulness. Posters mentioned feelings of guilt 32 times, and some described guilt for having negative feelings about caregiving and their loved ones.

I feel so guilty for the anger and resentment I feel. I always thought in our 50’s we would be traveling and enjoying ourselves and there is none of that. Whenever I think he is finally improving enough that we can do something enjoyable, he comes up with a new problem or complaint, which leads to more Dr. appointments, tests and procedures ... I feel sorry for myself and wonder what horrible things I did to deserve the life I have now.

[Caregiver Action Network]

Posters said they cried frequently 7 times. They described facing challenging decisions about their loved one’s care and the emotional toll of such choices.

My heart is breaking because I’m not sure if I’m ready to let him go but at the same time I know I can’t keep living like this, I’m feeling guilty cuz it feels like I’m giving up on him ... This is the hardest decision I’ve ever had to deal with but I know it’s the best decision, but then why do I feel so guilty?

[Mayo Clinic Connect]

Isolation was another common feeling described in 33 posts. Some lived in rural areas where there were few community groups or other social outlets. Others described a lack of connection to friends and other family members and difficulty engaging in employment.

My friendships are starting to be strained, because they don’t understand why “I’m hardly ever available” and quite frankly I’m starting to get bitter how they can just stay out as long as they want without worry. Romantic relationships are nonexistent, as most girls immediately become disinterested when they find out about my home situation. And in general I’ve found it harder to relate with other people, as my only social contact is in class or work.

[Caregiver Action Network]

Posters shared negative emotions 123 times and reported anger about the way their care recipient’s medical condition had impacted their lives, calling the situation unfair. Posters also described helping their care recipient deal with negative emotions 40 times, including anger about their diagnosis. Some posters detailed navigating emotional outbursts from the care recipient that were emotionally hurtful to the poster.

The last day that my Dad was home before going to a hospice facility, he kicked me when I was trying to clean him after a BM. He also punched at me and said “I’m going to beat you up!” Which wasn’t his normal personality- he was the sweetest, gentlest Dad usually. I know he didn’t feel good and he was embarrassed to need that much help, but I’m human and it still hurt my feelings.

[Facebook Empowering Caregivers Support Group]

A few posters expressed negative emotions toward themselves—saying they were “horrible” or a “monster” when describing difficult decisions involved in caregiving. This was often expressed along with frustration. One poster detailed how her husband had been put on hospice, then taken off, and lived more than 4 more years facing many health challenges. “WHY IS HE YET ALIVE[?]” she asked. Another described her internal wishes.

I pray every day my mother has a heart attack or a massive stroke.

[AgingCare]

While the majority of posts with emotion had a negative tone, 26 were positive. For example, some posters expressed fulfillment in their caregiving duties. Others said they were proud of the care they had been able to offer their loved ones, and some noted personal growth during the caregiving experience.

Over the years, I can see a huge change in myself. For one, I am more patient now. However, the goal line of ENOUGH patience keeps moving further away! I believe that my husband is providing me an opportunity to become a better person.

[Mayo Clinic Connect]

#### Connecting With Others in Similar Situations

Many posters expressed a desire to connect with others in similar situations, with web-based group support mentioned 301 times. As specific posters communicated more with one another, their comments took on a more familiar tone. A few even offered each other direct assistance.

If you are a man of faith, use it. If not, you may want to consider it and there are those who can help you with that. I’ll help you with that if I can. There is LOTS of help out there and there are LOTS of people that understand and are ready to be your friend and your lifeline.

[Caregiver Action Network]

Know that there are people that care. Like [name redacted] said, he will help, I will too, we are both reaching out to you, you are not alone.

[Caregiver Action Network]

Spiritual conversations appeared in posts 36 times. In some instances, the posters shared a positive spiritual statement as an initial post. In other instances, respondents promised to pray for the initial poster regarding the challenging situation or their care recipient’s medical condition.

As a caregiver myself to my husband, I’ve never felt more useless in my life. Feels like all you can do is watch and wait. There are days I’m afraid to go to sleep. I do pray for healing over your husband, and peace that surpasses all understanding for you. Bless you both.

[Mayo Clinic Connect]

Posters mentioned feeling lonely and shared their challenges with that across 20 posts. They brought up hobbies they no longer engaged in and not having time for friends or socialization. While respondents said that they had similar difficulty making time for themselves, they often prescribed self-care to one another.

Coping with the isolation of being a 24/7 caregiver is very challenging. It’s crucial to seek support from friends, family, or here with caregiving friends who understand your journey. Schedule regular breaks, explore local resources, delegate tasks, and set boundaries, which are essential for your well-being.

[Facebook Empowering Caregivers Support Group]

Posters also commiserated about the many tasks involved in caregiving. They described their day-to-day activities and the challenges associated with them. Respondents said they do the same things and appreciated knowing others were facing similar circumstances.

I feel like I am forever walking uphill. Frankly, one of the most helpful things I’m finding on here is that I am not alone. I don’t really need anyone to tell me what to do or fix this ... just to validate my feelings.

[Caregiver Action Network]

## Discussion

### Principal Findings

Our qualitative analysis of posts across 5 web-based caregiver forums showed that informal caregivers participate in these communities to share and receive moral and practical support. These forums functioned as a space for caregivers to disclose their daily struggles with those in similar circumstances. This was true across forums that offered more anonymity (AgingCare, Caregiver Action Network, and Mayo Clinic Caregivers Forum) but was also seen in the 2 Facebook groups that had the potential to be tied to caregivers’ personal lives.

Our research identified 5 key domains explaining why people posted on these caregiver forums. Within these themes were consistent comments about being heard and supported, highlighting the positive benefits that popular publicly accessible web-based caregiver forums can provide. Caregivers described putting significant time and effort into their caregiving, and their posts and stories showed how the role was a critical part of their lives. While the 5 domains we identified were similar to those found in prior research on web-based caregiver support that focused on particular topics related to caregiving, one website, or on caregivers of people with a specific diagnosis [17-30], this analysis demonstrated the consistency of these themes across several caregiver forums.

Many caregivers in this analysis experienced significant interpersonal tensions, including resentment and guilt when relating to the care recipient or other family members. Caregiving is often characterized by intense, intimate relationships with unexpected role changes and needs [41]. Research shows that spousal caregiving, in particular, can be more intense, with the informal caregiver experiencing feelings of depression and guilt [42,43]. Research also shows that caregivers caring for individuals younger than them may find more emotional, physical, and financial difficulties than their counterparts [44]. It is possible that caregivers anticipate caring for someone younger than them for a longer time, making it more difficult than caring for someone older. Similarly, our research indicates many caregivers are experiencing challenges with their relationships. These caregivers are reaching out on the web to vent and share their experiences with others, and this research shows that there are many places on the web where caregivers may find community among fellow caregivers likely to understand the relational intricacies and difficult situations they experience.

Informal caregivers used web-based forums to aid in navigating the maze of legal, financial, and health care systems. Previous research shows that informal caregivers experience significant difficulty identifying resources in the fragmented and complex health system and find a lack of consistency in access to community and home care [45]. In addition, there is insufficient community and health infrastructure for sharing resources with the caregiver community [45]. Caregivers use forums for aid related to logistical issues of caregiving in areas including legal, financial, and health care topics [23,32]. In our research, the problem-solving required to navigate these systems often stumped many caregivers and left them feeling frustrated and isolated. While respondents were frequently able to provide helpful steps to guide the original poster toward the desired outcome, our findings support the need for additional informal caregiver support toward navigating the necessary solutions within health and community systems.

In our study, informal caregivers expressed persistent concerns about managing practical daily matters. The range of such daily demands on caregivers is vast, with tasks such as feeding and bathing, paying bills and shopping, caring for property and financial matters, and offering encouragement and companionship. Informal caregivers who help with a greater number of activities of daily living or independent activities of daily living experience more caregiver burden [46]. Additionally, caregiving often includes more medical-focused tasks; yet, practical daily tasks were the dominant type mentioned by posters in our study. Research indicates that support with medical-focused tasks from effective interventions and health care providers can help informal caregivers improve their caregiving experience [47,48]. Web-based caregiver forums are likely a better source for advice for practical daily tasks than medical-focused tasks, given that providing advice on the latter involves having medical expertise.

Every web-based forum we reviewed included numerous caregivers who expressed gratitude and encouragement related to connecting with others walking a similar path in life. Whether it was offering a step-by-step suggestion on how to address a problem, thanking each other for sharing, or simply saying, “Hang in there,” it was clear that the posters in our study appreciated the connection the forum provided. Similarly, a previous study analyzing web-based caregiver forums during COVID-19 identified a common theme of “celebrating” the virtual way of life [19]. In that study, posters highlighted the sense of community in the forum and how engaging with the site had helped them with caregiving during the pandemic.

Our analysis highlighted web-based forums as places where caregivers shared thoughts and sensitive caregiving details that could be considered socially inappropriate—things they might not say in an interview or to someone they know—such as comments on family disagreements, personal hygiene, and the caregiver asking “Why is he yet alive” about her spouse. This finding is consistent with another study that found participants in web-based caregiver groups felt safe sharing thoughts that were not socially acceptable [49]. Additionally, research shows that reciprocity, altruism, and empathy have a greater influence on knowledge sharing on web-based forums for nonhealth professionals than health professionals [50]. These motivations help explain the candid statements made by posters in our study and suggest that the informal caregivers want to help one another.

Many of the posts on the caregiver web-based forums in our analysis were emotionally charged, and posters seemed to use these communities as a way to vent negative emotions. Spousal caregivers, in particular, have been found to share more negative emotion and anxiety than other informal caregivers [22]. Furthermore, previous research found that informal caregivers who share negative aspects of caregiving on the web may ask others to confirm that their feelings are shared [32]. One reason that sharing frustrations on the web may help informal caregivers is that the negative emotions can be posted as soon as they experience the frustration [51].

Our study comes after the COVID-19 pandemic, a challenging time for informal caregivers, who often experienced increased burden from illness for themselves and their care recipient, had to navigate pandemic-related policies like medical office closings, and experienced social isolation [52,53]. Web-based discussion forums provided a medium for connecting with others and gaining information during the pandemic. Older adults, in particular, became more comfortable with using the internet and other technologies during the pandemic, with digital connectivity playing an increased role in their lives even after the pandemic [54]. With many older adults caring for spouses, siblings, and friends, access to web-based caregiver forums is a tool that aging adults, for one, can continue to give and receive support.

### Limitations

Our study had some limitations. Due to the anonymity of the web-based forum posters, information on their descriptive characteristics could not be obtained. Additionally, the web-based caregiver forums we examined were based in the United States. While the internet is available globally, it is likely that the posters were primarily based in the United States and experiencing caregiving in that country. This may limit the generalizability of the study findings on a global scale. For example, many health system challenges highlighted in the study are specific to those in the United States. In addition, this research captures caregivers’ posts at one point in time and may not be generalizable in the future, as the caregiver landscape changes over time. Finally, it is possible that some posters were not informal caregivers, even though the content seemed to be posted by informal caregivers.

### Conclusions

This paper contributes meaningfully to the current state of the art by providing a rich understanding of informal caregiving through an innovative lens, that of web-based forums. From this work, researchers and practitioners can be better informed about the needs of informal caregivers beyond what such caregivers may mention in interviews or medical appointments. By illuminating the lived experiences shared in web-based forums, the study advances knowledge about caregiving.

Informal caregivers play an essential role in society. Each year, informal caregivers in the United States provide as much as 36 billion hours of care to adults with limitations in daily activities, and such caregiving is linked to significant emotional, physical, and financial burdens to informal caregivers [55]. Since most informal caregivers have internet access [8], accessing web-based discussion forums is a low-barrier method for them to reach a community of people who may be experiencing similar emotions and challenges. With the number of American people aged 65 years and older projected to jump from 53 million [1] to nearly 95 million by 2060, the need for informal caregiving is projected to grow substantially [56]. Our study provides insights into how informal caregivers use web-based caregiver forums and can inform health care providers and community groups who provide information, education, and support to patients and their informal caregivers. In summary, web-based caregiver forums enable informal caregivers to use modern technology to help meet their support needs and, in the process, help to show them that they are not alone in facing caregiving challenges.
